# 757 A Patient Inclusion Criteria Algorithm for New Burn Centers Seeking to Implement a Burn Registry

**DOI:** 10.1093/jbcr/irac012.310

**Published:** 2022-03-23

**Authors:** Amanda Guilford, Jeneva Garland, Ransom Wyse, Lori Harbour, Rebecca Smith, Monica Bregman, Samuel W Jones

**Affiliations:** Chippenham Hospital, Richmond, Virginia; Center for Trauma and Acute Care Surgery Research, HCA Healthcare, Nashville, Tennessee; Center for Trauma and Acute Care Surgery Research, HCA Healthcare, Nashville, Tennessee; Center for Trauma and Acute Care Surgery Research, HCA Healthcare, Nashville, Tennessee; Chippenham Hospital, richmond, Virginia; Chippenham Hospital, Richmond, Virginia; Chippenham Hospital, Richmond, Virginia

## Abstract

**Introduction:**

Data submissions to the NBR are based upon the standardized medical definitions and generalized guidance on patient inclusion criteria found in the ABA Burn Care Quality Data Dictionary. The guidance is complex, often leading to confusion in determining proper patient identification. This is most problematic for new burn centers seeking to implement a burn registry. Therefore, at our burn center, we designed and implemented an algorithm based on a thorough literature review and ABA guidance to improve the accuracy and efficiency of patient identification.

**Methods:**

A review of the literature was conducted using the PubMed database. In addition, utilizing the ABA’s multitude of resources including, the Burn Care & Quality Platform (BCQP) Data Dictionary 2020 version 1.1 patient inclusion criteria statements, a BCQP Q&A session on admission information, and Quality & Registry Community discussion boards, the following algorithm was developed by the compilation and simplification of the definitions provided for burn and non-burn injury patient inclusion criteria.

**Results:**

A thorough literature review produced no articles that address NBR inclusion criteria. According to these documents, all patients admitted to the hospital for treatment of an acute burn or soft tissue wound should be included in the burn registry; however, a BCQP Q&A session expressed the Burn Service must provide medical care in order for a patient to be included in the registry. Furthermore, the BCQP Data Dictionary outlines patient inclusion based on a hospital length of stay >24 hours, a surgical operation, or expire at a hospital facility for registry inclusion. Observation patients and non-burn patient consults are excluded from the NBR. The ABA community discussion board revealed many centers only count and track non-burn injury admissions to the burn center service line. The compilation of information resulted in the development of a standardized algorithm defining a patient for registry inclusion.

**Conclusions:**

A comprehensive review of the ABA resource documents was compiled to clarify the patient inclusion criteria for data submission to the NBR. The BCQP Data Dictionary 2020 version 1.1 patient inclusion criteria statements, the collaborative Ameriburn communities, and the BCQP Q&A session influenced the design of the clarification algorithm for patient identification to assist burn centers seeking to accurately and most efficiently implement a burn registry. Further research should be conducted on the utilization of the algorithm in comparison to other burn centers.

